# Hip Anatomy and Ontogeny of Lower Limb Musculature in Three Species of Nonhuman Primates

**DOI:** 10.1155/2011/580864

**Published:** 2011-07-19

**Authors:** Jeremy J. Baker, Katherine J. Searight, Madeliene Atzeva Stump, Matthew B. Kehrer, Colleen Shanafelt, Eric Graham, Timothy D. Smith

**Affiliations:** ^1^Drayer Physical Therapy Institute, Austintown, OH 44515, USA; ^2^School of Physical Therapy, Slippery Rock University, Slippery Rock, PA 16057, USA; ^3^Medical Scientist Training Program, Carver College of Medicine, The University of Iowa, Iowa City, IA 52242, USA; ^4^HealthPoint, Wooster, OH 44691, USA; ^5^Department of Anthropology, University of Pittsburgh, Pittsburgh, PA 15260, USA

## Abstract

The hip region is examined to determine what aspects of musculoskeletal anatomy are precociously developed in primate species with highly specialized modes of locomotion. Muscles of the hind limb were removed and weighed in each specimen, and the hip joint of selected specimens was studied in stained serial sections. No perinatal differences among species are evident, but in adults, the hip joint of *Galago moholi* (a leaping specialist) appears to have proportionally thick articular cartilage (relative to the subchondral plate) compared to two species of cheirogaleids. Muscle mass distribution in the hind limbs confirms previous observations that the quadriceps femoris muscle is especially large in *Galago* (in percent mass of the entire hind limb), while the hip region is smaller compared to the more quadrupedal cheirogaleids. Across age groups, the species with the least specialized locomotion as adults, *Cheirogaleus medius*, shows little or no change in proximal to distal percentage distribution of muscle mass. *Galago* has a larger percentage mass gain in the thigh. We suggest that muscle mass gain to specific limb segments may be a critical milestone for primates with extremely specialized modes of locomotion.

## 1. Introduction

The hip region of primates varies considerably in morphology and relative dimensions, and previous investigations have identified correlates to positional and locomotor behaviors [2–4] (and see Anemone, 1993 [5] for review). Musculoskeletal specializations differentiate primates that employ certain locomotor patterns, such as vertical clinging and leaping, from other primates. For example, leaping specialists (such as indriids, tarsioids, lepilemurids, and some galagids) have a more proximally positioned lesser and third trochanters [2, 5]. These primates also have relatively large muscle compartments for hip extensors, knee extensors, or ankle plantarflexors, an adaptation related to emphasis on hind limb propulsion via leaping.

In comparing leaping versus quadrupedal primates, certain aspects of the anatomy of the hip have received relatively little scrutiny. Joint microanatomy in primates has received little attention (but see Dewire and Simkin, 1996 [6]). We are aware of no microanatomical studies of articular cartilage (AC) on strepsirrhine taxa. Ossification patterns of joints are well studied in haplorhines [7–10], but strepsirrhines are understudied by comparison. Generally, development of the hip has received far less attention than other anatomical regions. Muscular anatomy of the limbs has been well studied [4, 11, 12] but rarely regarding ontogeny [1, 13, 14]. Recently, we investigated the ontogeny of muscle mass distribution in the hind limb of primates that use primarily leaping modes of locomotion versus arboreal or terrestrial quadrupedalism [1]. Again, in this study, less emphasis was placed on the hip due to difficulty in dissecting the region in the smallest infants. 

In the present study, we expand the scope of a previous investigation from our laboratory [1]. Musculoskeletal structure of the hip region is studied in sample of perinatal and adult strepsirrhine primates that differ in locomotor behaviors. Specifically, distribution of muscle mass in the hip and other segments, as well as microanatomy of the hip joint, are studied in arboreal quadrupeds and a leaping specialist. These data are examined to determine what aspects of musculoskeletal anatomy are precociously developed in primate species with highly specialized modes of locomotion. 

## 2. Materials and Methods

### 2.1. Sample and Species Characteristics

The sample included eight *Galago moholi *(4 adult, 4 perinatal), nine *Cheirogaleus medius* (4 adult, 5 perinatal), and nine *Microcebus murinus* (6 adult, 3 perinatal). Muscle mass data described in this study incorporate some previously published data. Two of the adult and three perinatal *C. medius *were previously measured [1] and combined with newly measured specimens (2 adult, 2 perinatal) to achieve a larger sampling of the species. All muscle mass data on *M. murinus* were previously published, and are graphically compared to the other species.

The species under study were selected based on their contrasting locomotor patterns, as described by Walker [15]. The southern lesser bushbaby (*G. moholi*) uses vertical clinging and leaping patterns of locomotion and uses upright locomotion on terrestrial substrates. Although some galagids employ more quadrupedal behaviors than others, *G. moholi *resembles *G. senegalensis *in its heavy reliance on leaping behaviors and vertical posture [16]. The fat-tailed dwarf lemur (*C. medius*) and gray mouse lemur (*M. murinus*) use arboreal quadrupedalism for locomotion. However, *M. murinus *is described to employ the most leaping behaviors among the cheirogaleids [15]. 

In addition to behavioral differences, a comparison of the differences in their development may help with interpretation of our findings. Based on behavioral and life-history observations, *G. moholi *and the cheirogaleids studied here have key differences in ontogeny. Some similarities do exist. In all species, infants are described to perfect their locomotor behaviors over the course of months [16–18]. Infant *C. medius *are cached in nests for about two weeks, except when carried orally [19]. *G. moholi *(and *G. senegalensis*) similarly show a preference to stay in the nest box for 1-2 weeks [16, 20] but are described to be very active [16]. Some milestones, such as weaning, are achieved earlier in the cheirogaleids than *G. moholi* (and also compared to* G. senegalensis*) [21].

During infancy, the comparison seems more starkly different. For example, newborn *M. murinus *are described as more altricial than *G. moholi* (e.g., the former born with eyes closed, the latter, eyes open) [16, 22]. *G. moholi *may be more precocious in development of its locomotor specialty. Doyle [16] asserts captive infants of this species are extremely active in the nest and can make small jumps within 10 days although they continue to become stronger leapers during the first 2 months. In contrast, *M. murinus *exhibits leaping behaviors after about 3 weeks, and the first movements by *C. medius *are described as similar to adults but clumsy (see review of cheirogaleid locomotor ontogeny by Atzeva et al. [1]). A study of captive *C. medius *noted “jumping and running” by postnatal days 27 to 30, and all adult locomotor behaviors were seen by day 40 [18]. Based on these descriptions, we assume that cheirogaleids are somewhat less precocious than *G. moholi *in the development of locomotor behavior. These present study, in part, assesses whether hind limb musculoskeletal characteristics differ based on preciousness.

### 2.2. Investigative Methods

All specimens were acquired as cadaveric remains from the Duke Lemur Center, except one perinatal *Galago* cadaver (courtesy of L. Martin). All specimens died of natural causes and most were immersed in formalin or frozen and then immersed in formalin. One perinatal *G. moholi *was fixed in 70% ethanol. The captive primates were maintained in a seminatural environment that allowed the use of their preferred pattern of locomotion without restriction. All specimens were available as a result of natural deaths in captivity. Perinatal and older infant cadavers were stillborn or postnatal deaths from 0 to 15 days postnatal age. No grossly obvious pathologies, such as limb or limb joint deformities, were found among perinatal specimens. 

The same protocol was used for the dissection and weighing of all specimens. Dissection protocol included removal of skin and connective tissue to expose underlying limb musculature. Infants were dissected with the aid of a dissecting microscope. Once the underlying musculature was revealed, muscles were identified and removed. 

After removal, muscles were grouped according to function and were weighed by a single investigator. Weights were obtained for the following functional groups: hip extensors, hip adductors, hamstrings, quadriceps, superficial ankle flexor, deep ankle flexors, hip external rotators, anterior compartment of the leg, and the lateral compartment of the leg (Table 1). Muscles responsible for several movements were weighed individually (e.g., sartorius). Certain muscle groups (such as the external rotators) were too small to reliably dissect in some infants. This prevented certain analyses, such as functional groupings of hip muscles in infants. Intrinsic muscles of the hands and feet were difficult to remove and were excluded from the data. Some individual muscles in infants were found to have a mass near the 0.001 level of accuracy. In these cases, the entire functional muscle compartments were removed and weighed as a unit weighed as a unit. All muscles were removed from bone and connective tissue and blotted dry with paper towel prior to weighing. Muscle masses were obtained with a Mettler AJ100 scale and were recorded to the nearest 0.001 g for infants and to the nearest 0.01 g for the adult specimens. Muscles/muscle groups were weighed twice and the average of the two recordings was used. In cases of measurement discrepancy exceeding 10%, a third measurement was taken and the outlier thrown out. 

The present study employs relatively small samples. However, this sample is larger than previous studies on hind limb muscle masses in prosimian species and allows nonparametric statistical tests. Since the infant samples were smaller samples and it was not possible to weigh muscles in functional groups, the statistical analysis is limited to adult sample. Data on *Galago, Cheirogaleus *and *Microcebus *(from Atzeva et al., 2007 [1]) were compared regarding the percentage of muscle mass for hind limb propulsion, including hip extensors, knee extensors, and ankle plantarflexors. Data were compared between groups using a Kruskal-Wallis one-way analysis of variance test. Differences between species were then assessed using a Mann Whitney *U*-Test. Statistical significance was determined using a sequential Bonferroni correction [24]. We regarded each muscle group and an independent series of tests, with three post hoc tests to determine which pairs were different. With our threshold at *P* ≤ .05, the pair with the lowest *P* value in the Mann Whitney *U*-Test was considered significant at *P* ≤ .017, followed by *P* ≤ .025 and *P* ≤ .05.

Joint histology was studied in a subset of this sample. Hip joint tissues were extracted in all adults except two dwarf lemurs (which are now part of the collection of the Carnegie Museum, Section of Mammals). One perinatal specimen of each species was used to establish degree of ossification at the hip joint. In addition, a single perinatal *Galagoides demidoff *was available for study to broaden the comparative perspective. 

Following muscle dissection, the hip joint was removed by cutting across the iliac blade, through the pubic symphysis, and across the surgical neck of the femur. The hip joint was decalcified using a sodium citrate-formic acid solution (duration: approximately two weeks for infants; approximately one and a half months for adults). Following decalcification, joints were briefly returned to 10% buffered formalin and processed by graded dehydration, clearing in xylene, and paraffin embedding. During paraffin embedding, joints were positioned in the embedding tray so that the femur would be sectioned, as nearly as possible, in the frontal plane. The hip joints were then serially sectioned at 10 to 12 *μ*m using a Leica rotary microtome, and every tenth section was stained with hematoxylin-eosin for general structural examination. 

Selected sections were stained using two procedures to identify connective tissues, Gomori trichrome and Picro-Ponceau. Using either of these procedures, highly collagenous tissues (such as bone) are more densely stained than cartilage, thus allowing identification of the boundary between the subchondral bone and AC. These preparations were used for a preliminary analysis of AC thickness in the adult primates. Sections that appeared to be in the mid-level through the femoral head were photographed at ×25 to ×50 using a Leica DMLB photomicroscope with a DKC-5000 Catseye Digital Still Camera System (Sony Electronics Inc., Montvale, NJ, USA). Images were then opened using ImageJ 1.43 (NIH). For measuring AC thickness, the joint surface was measured at different locations. This was undertaken because all parts of AC do not exist in an identical biomechanical regimen [25], and no single locus can be assumed to reflect average AC thickness. Our method loosely follows Mork et al. [26] who assessed the cartilage of the temporomandibular joint in three zones. Since our measurements were based on subchondral bony landmarks, we could not use identical positions with these regions (e.g., specific positions along arc length), because trabecular attachments obscure the deepest extent of subchondral bone. Using the ×25 micrographs, the joint surfaces were examined by microscopy to locate superior, middle, and inferior thirds. Then, higher magnification (×50) images of each third were photographed. Measurements were taken near the center of each region, avoiding loci where trabeculae interfaced with the subchondral bone. In each third, two measurements were taken, each along a line that measured the depth from the hip joint cavity to the marrow cavity of the femur. First, the image was calibrated in pixel dimensions to a stage micrometer that was photographed at ×50. Then, the distance from the surface of the AC (facing the joint cavity) to the deepest extent of the subchondral plate (facing the marrow cavity) was measured. Next, following the same line, the distance from the joint surface to the interface of the AC and subchondral plate was measured. By subtracting these two dimensions, subchondral plate thickness was computed. 

## 3. Results

### 3.1. Musculature and Muscle Mass Distribution

**Figure 1:**
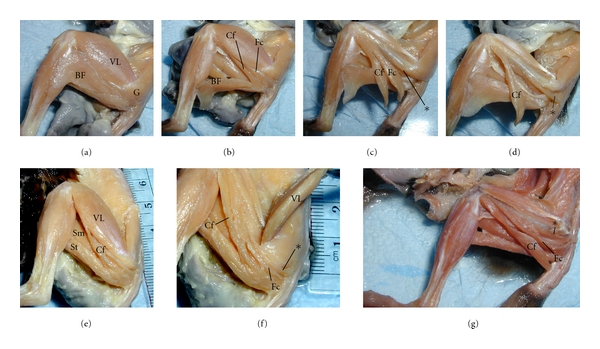
Gross organization of the hip musculature in *Microcebus murinus, *lateral view of left hind limb. (a)–(d) show a single adult specimen at various stages of dissection. (a) The thigh musculature is intact, showing the large vastus lateralis (VL) balanced by a large biceps femoris (BF). These muscles partially obscure the posterior portion of gluteal musculature (G). (b) BF removed, exposing the caudofemoralis (Cf) and femorcoccygeus (Fc). (c) When the Fc is removed, additional musculature remains (*), running parallel, but deep to the Fc. (d) This muscle is shown with its sacral point of origin removed; the Fc has been removed to better emphasize *; a different adult is shown in (e)–(f) with the BF removed (e), and VL resected (f). (g) A third adult is shown, in an advance stage of dissection, emphasizing *, which may be an accessory head of the Fc. Sm, semimembranosus; St, semitendinosus.

Gross muscular organization of the hip is not considered in great detail, since almost no novel aspects could be observed. The hip musculature of *G. moholi *showed no notable departure from the description of hip musculature of *G. senegalensis* by Stevens et al. [27]. In the cheirogaleids, hip musculature closely resembled previous descriptions of *M. murinus *and *C. major *by Jouffroy [11]. However, it is noted that the gluteus superficialis posterior is more complex in the *M. murinus *specimens than noted in previous reports or compared to *C. medius*. After superficial muscles are resected (Figures 1(a) and 1(b)), and the caudofemoralis and femorococcygeus is removed from their origin point (Figure 1(c)), a smaller muscle is visible in most of our *M. murinus *specimens, running in parallel to the femorococcygeus (Figures 1(c)–1(g)). The muscle is differentiated from the femorococcygeus in all but one of the *M. murinus*. This small muscle has an ischial origin and insertion to the femoral shaft (Figure 1(c)) as seen in the femorococcygeus, but it has a deeper, more distal origin and a more proximal insertion.

**Figure 2:**
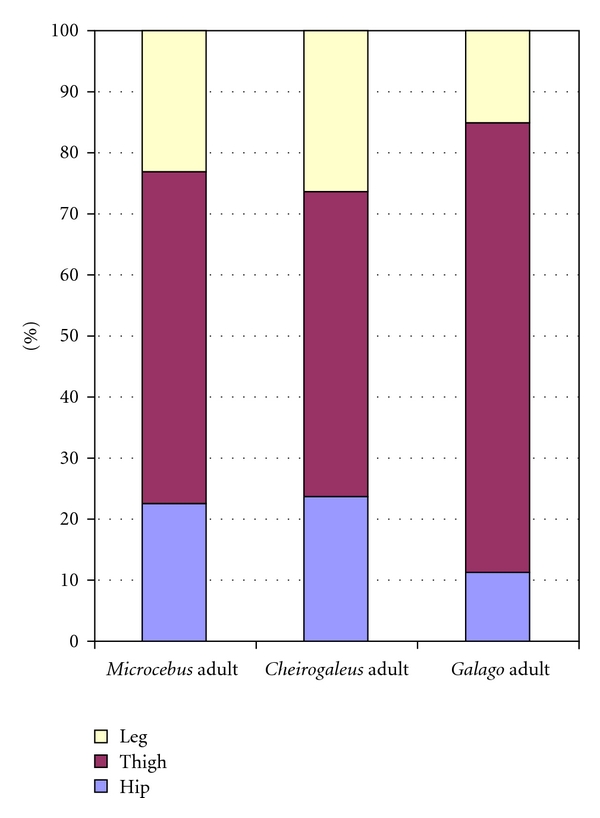
Comparison of hind limb muscle mass distribution among segments (excluding intrinsic foot muscles) in three species of primates at adult age. Data for *M. murinus* from Atzeva et al. [1].

**Figure 3:**
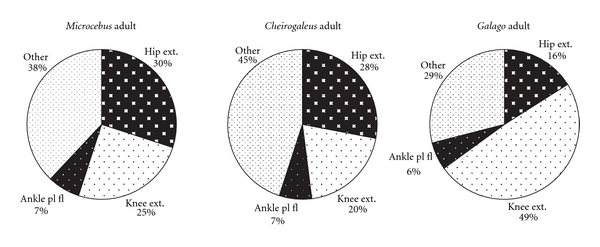
Distribution of hind limb musculature in three species of primates at adult age. Percentage mass of functional groups is indicated (i.e., hamstring mm included with hip extensors). Graphs based on mean muscle mass presented in Table 1; data for *M. murinus* from Atzeva et al. [1].

**Figure 4:**
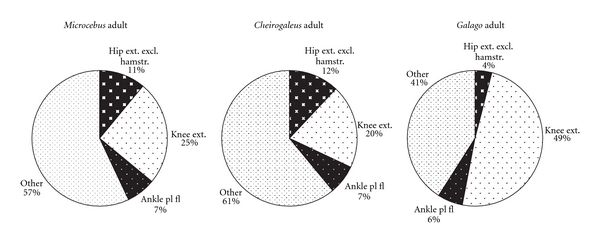
Distribution of hind limb musculature in three species of primates at adult age. Percentage mass in limb segments is indicated (i.e., hamstring mm excluded from hip extensors). Data for *M. murinus* from Atzeva et al. [1].

The relative distribution of all hind limb musculature in adult samples is shown in Figures 2 to 4 (graphs in Figures 3 and 4 are modeled after Demes et al. [4]). Data on the two species dissected for this study are compared to findings on *M. murinus *(source: Atzeva et al. [1]). For the lower limb, excluding the intrinsic foot muscles, most muscle mass comprises thigh musculature in all species (Figure 2). The thigh muscle mass is proportionally greatest in *G. moholi *(74%) and least in *C. medius *(50%). In the cheirogaleids, muscle masses of the leg and hip are similar (close to 25%), whereas *G. moholi *has a notably small percentage (11%) distributed to the hip. When considered as functional groups according to joint motion (Figure 3) or functional groups within each segment (Figure 4), cheirogaleids and *G. moholi *show differing organization of mass. Overall, the muscles involved in propulsion comprise 71% of hind limb muscle mass in *Galago* compared to 62% in *Microcebus *and 55% in *Cheirogaleus *(Figure 3). Both cheirogaleids possess a proportionately large percentage of hip extensor muscle mass compared to the *G. moholi*. In the latter, knee extensors are by far the largest percentage mass for hind limb propulsion (Figure 3). Kruskal-Wallis one-way analysis of variance tests revealed significant (*P* < .05) differences among the three species for percentage hip extensors, percentage knee extensors, and percentage ankle plantarflexors (Table 2). Mann-Whitney *U*-tests revealed that there are significant (lowest threshold at *P* < .017, following sequential Bonferroni corrections) intragroup differences in percentage hip flexors (*Galago < Microcebus*), knee extensors (*Microcebus *< *Galago*), and ankle plantarflexors (*Galago < Microcebus*).

In Figure 4, hamstrings are excluded from the hip extensor mass, thus emphasizing the gluteal extensors (gluteus superficialis posterior, gluteus medius, and gluteus minimus). In all three species, gluteal extensors represent the least percentage mass for limb propulsion, but they are especially minimal in *G. moholi.*

**Figure 5:**
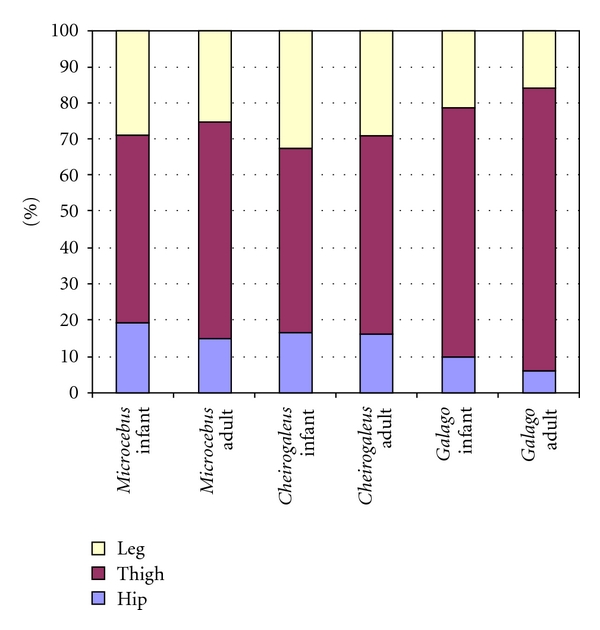
Comparison of hind limb muscle mass distribution among segments (excluding intrinsic foot muscles) in three species of primates: age comparisons. For this graph, iliopsoas m. and external hip rotators are excluded because they were not measured in all perinatal samples. Data for *M. murinus* from Atzeva et al. [1].

**Figure 6:**
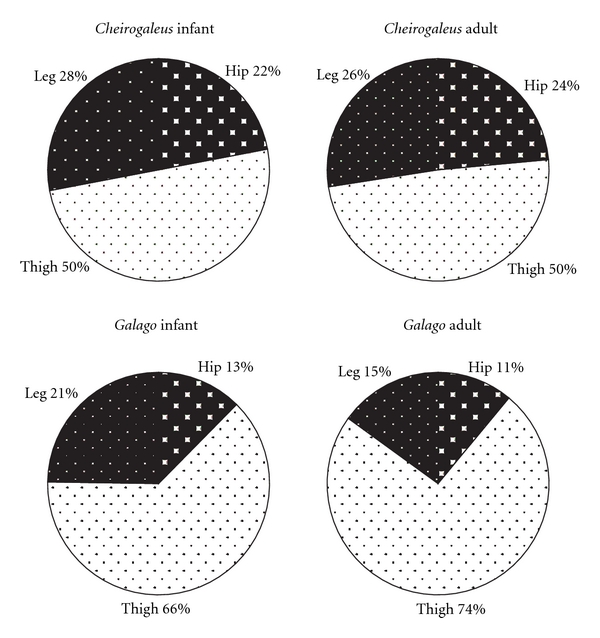
Comparison of hind limb muscle mass distribution among segments (excluding intrinsic foot muscles) in *C. medius *and *G. moholi*: age comparisons. For this graph, iliopsoas m. mass is included in the hip. Hip percentage in infant *C. medius* should be regarded with caution, since the iliopsoas muscle could only be weighed in one specimen.

Across ages, all three species show a relative shift in muscle mass toward the thigh, that is, the thigh increases to a greater extent than other segments (Figure 5). This mass shift is more pronounced in *G. moholi *(with a 7% increase from perinatal to adult) and *M. murinus *(9% increase) than in *C. medius *(4% increase). Since iliopsoas could not be measured in perinatal specimens of *M. murinus* [1] the percentage comparisons in Figure 5 should be viewed with some caution. A more complete comparison of age changes in muscle mass distribution is possible between *C. medius *and *G. moholi,* in which only hip external rotators are excluded from percentage calculations (Figure 6). When iliopsoas is included, *C. medius* appears to change very little in mass distribution from perinatal to adult samples; a percentage mass shift to the thigh is not detected at all. In *G. moholi*, the percentage mass shift to the thigh appears slightly greater (8%), and there is a proportional decrease in leg muscle mass (6%).

### 3.2. Joint Microanatomy and Ossification Centers

**Figure 7:**
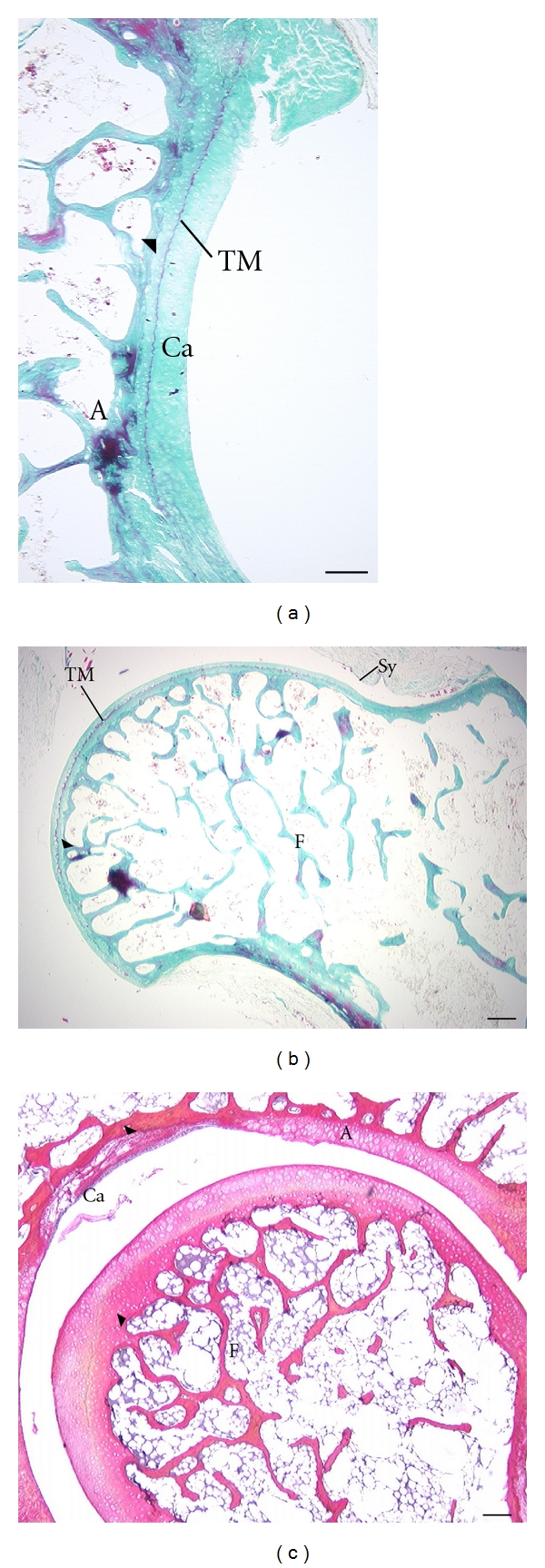
Articular cartilage in adult cheirogaleids, acetabular (A) and femoral (F) surfaces are shown. Superior aspect is at the top of the image. Arrowheads indicate the junction of the subchondral plate and articular cartilage. (a, b) *C. medius *(same specimen); (c) *M. murinus *(2 different specimens). Note the difference in articular cartilage thickness of the acetabulum compared to the femoral head in *C. medius *(a, b). This is most apparent when viewing the extent of cartilage that is between the tidemark (TM) and the joint cavity (the TM is the line separating the deeper mineralized cartilage matrix from the more superficial matrix.) There is less disparity in articular cartilage thickness between the joint surfaces in *M. murinus *(c). Ca: joint cavity; Sy: synovial membrane. Stains: a, b: Gomori trichrome preparation; c: Picro Ponceau. Scale bars: a, c, d: 300 *μ*m; b: 200 *μ*m.

**Figure 8:**
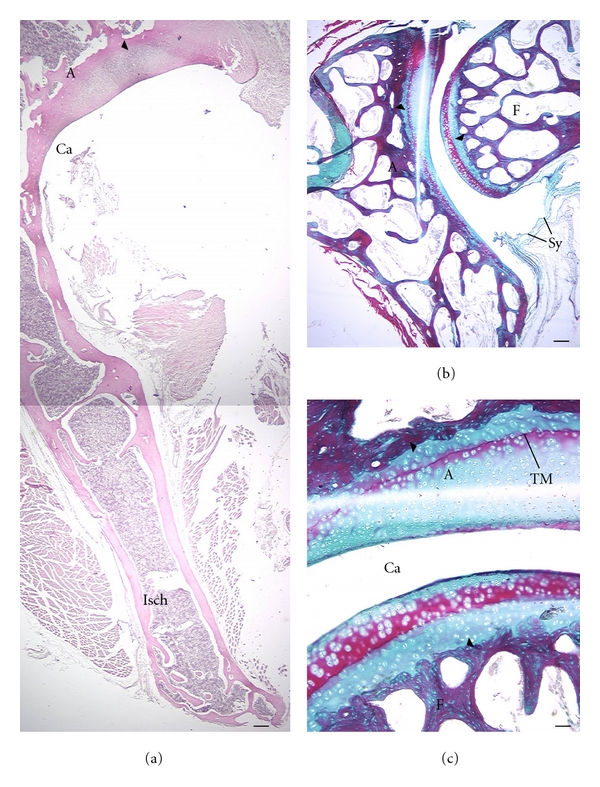
Articular cartilage in adult *G. moholi*. Superior aspect is at the top of the image, except in (c) (superior is to the left). Arrowheads indicate the junction of the subchondral plate and articular cartilage. (a) The proportionally thick articular cartilage of the acetabulum (A) is shown; (b, c) show a different specimen; (b, d) revealing proportionally thick articular cartilage over the acetabulum and femur (F). Ca: joint cavity; Isch: ischium; Sy: synovial membrane. Stains: a, hematoxylin eosin; b, c: Gomori trichrome preparation. Scale bars: a, b: 300 *μ*m; c: 200 *μ*m; TM: tidemark.

Articular cartilage thickness appears to differ more between the acetabulum and femur in *C. medius *(Figures 7(a) and 7(b)) compared to *M. murinus *(Figure 7(c)). Thickness of the AC appears proportionally greater in *G. moholi *(Figure 8) compared to cheirogaleids. 

Analysis of AC thickness supports these qualitative observations. These quantitative results should be regarded as preliminary since only one of the two *C. medius *and three of the four *G. moholi *were suitable for measurements (the others had indistinct deep or superficial limits of the AC). In *G. moholi*, average AC thickness of the acetabulum is more than 2-fold greater than that of the femoral head (Table 3). A similar, though less pronounced disparity, is observed in *C. medius. * In *M. murinus*, this relationship is not observed; average femoral AC thickness is slightly greater than that for the acetabulum (Table 3). Thickness of the subchondral plate follows the same trend among species (Table 3). However, ratios of AC thickness/subchondral plate thickness are highest for both joints in *G. moholi *compared to the cheirogaleids (Table 3). 

**Figure 9:**
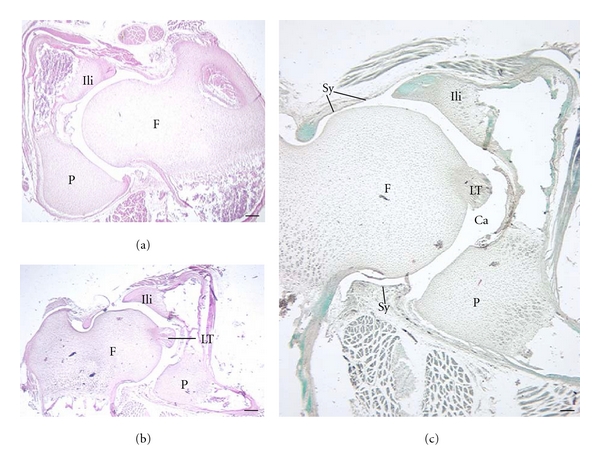
Ossification center in 2 perinatal cheirogaleids. Superior aspect is at the top of the image. *C. medius *(a) and* M. murinus *(b, c) are shown. The proximal epiphysis of the femur (F) and the iliac (Ili) and pubic (P) centers of ossification are at least partially cartilaginous. Ca: joint cavity; LT: ligamentum teres; Sy: synovial membrane. Stains: a, b, hematoxylin eosin; c: Gomori trichrome preparation. Scale bars: a, b: 200 *μ*m; c: 300 *μ*m.

**Figure 10:**
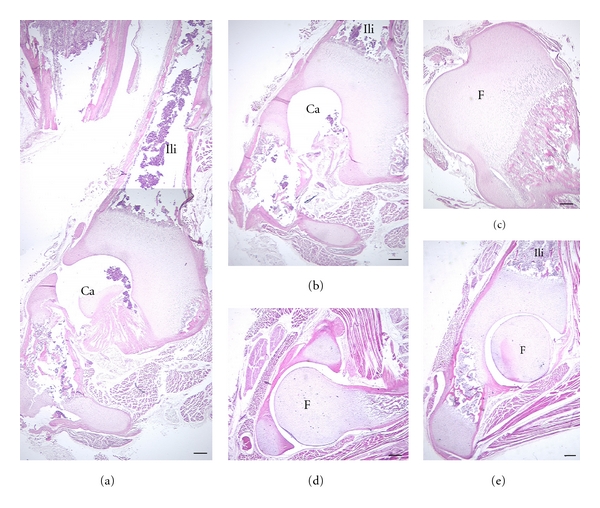
Ossification center in 2 perinatal galagids. Superior aspect is at the top of the image. *G. moholi *(a)–(c) and* G. demidoff *(d, e) are shown. Note similar extent of ossification of the femur (F) and portions of the os coxa compared to cheirogaleids (Figure 9). Stains: hematoxylin eosin. Ca: joint cavity. Scale bars, 300 *μ*m; Ili: ilium; A: acetabular joint space.

Sections of selected perinatal hip joints suggest no appreciable differences among species (Figures 9 and 10). In all cases the secondary ossifications center at the proximal femur is cartilaginous (Figures 9(a)–9(c) and 10(c)–10(e)). The os coxae show ossification in all species. Cartilage closely adjacent to the joint remains largely unossified (Figures 9, 10(a), 10(b), 10(d), and 10(e)). But primary ossification centers such as the iliac blade are well ossified (Figure 10(a)). 

## 4. Discussion

### 4.1. Gross Anatomy of the Hip

 Gross organization of hip musculature has been well described previously [5, 11, 27, 29]. The gross descriptions of muscular anatomy offer little additional insight to previous descriptions, except for a possible accessory muscle. As the muscle lies on the extensor side of the hip joint, this might best be considered a deep head of the femorococcygeus. The remainder of this discussion will be devoted to more novel results.

### 4.2. Microanatomy of the Hip Joint

Although joint morphology has been subject to great scrutiny by students of primate anatomy (e.g., [30–32]), few studies have considered joint microstructure. The relatively recent increase in availability of high-resolution, nondestructive methods, such a computed tomography, seems to make the topic of great potential interest. The thickness of the subchondral plate in primates was studied using computed tomography by Dewire and Simkin [6]. These authors found little variation in the thickness of the subchondral plate in the femur but significant variation in subchondral plate thickness of the acetabulum (increased thickness with increased body size among primates). An unknown in their study, unavailable using computed tomography, is the thickness of AC across primates.

Micro-MRI, currently of great promise for studying osteoarthritis [33], may provide a viable avenue for studying AC in cadaveric primates. The destructive methods used to study AC in this study are admittedly an undesirable means to produce large samples of nonhuman primates for quantitative analyses. As a result, our sample is too small for a quantitative analysis. At present, however, no other method allows the same resolution to describe AC in minute detail. Thus our preliminary observations may provide insight for future studies. 

In two of the species (*C. medius *and *G. moholi*), the AC thickness of the acetabulum was thicker than that of the femur, which could reflect development of concave and convex surfaces under different stress histories [34]. This relationship was not apparent in *M. murinus, *however. A difference observed between cheirogaleids and *G. moholi *was the greater thickness of AC in the latter. Paraffin sectioning can produce distortions that might alter the apparent thickness of tissues. For example, slight deviations in cutting plane could hypothetically make AC appear thicker from surface to subchondral plate if sectioning is not at a right angle to the subchondral plate. While consistent cutting planes can be hard to achieve with paraffin blocks, there is a strong basis for an assertion that the results reflect true species differences. First, the range of AC thickness of *G. moholi *specimens exceeded that of the other two species; in the case of the acetabular AC, there is no overlap with the other species (Table 3). Secondly, the AC/subchondral plate thickness ratios are highest in *G. moholi. * Thus, the greater thickness of the AC is *proportional *to the subchondral plate. Presumably, planar distortion would affect not just the AC, but the subchondral plate as well. 

Variations in articular cartilage thickness have been related to body weight in humans, where it has been suggested larger individuals have thicker AC in lower limb joints [35], and some scaling of AC thickness to body mass could be inferred by comparing *M. murinus *to the other species. AC thickness has also been related to anisotropic properties of the tissue, based on its tendency to grow based on region-specific response to the magnitude of hydrostatic pressure due to compressive loading [31]. That species differences relate only to body size seems unlikely since *G. moholi *is not greatly larger than *C. medius*. Thus, species differences may also relate to the contrasting locomotory behavior of cheirogaleids compared to *Galago*. At the present time, a broad perspective on primate AC is lacking, due to the lack of similar studies. An analysis of a larger taxonomic sample of primates, optimally with nondestructive methods, is needed to establish diversity in joint microanatomy as well as functional correlates.

### 4.3. Distribution of Muscle Mass

These findings also provide an update on results presented by Atzeva et al. [1]. That study focused on ontogenetic changes in limb muscle mass distribution in cheirogaleids and other primates, with a limited discussion of hip musculature, since it could not be reliably dissected in perinatal specimens. By including the hip muscle mass in the present study, a clearer view of the entire limb muscle mass distribution is provided here. 

The results of the present study confirm certain previous findings on muscular specializations of prosimian primates, for example, the well-developed thigh muscle mass in adult lesser galagos. In this regard, our findings on *G. moholi* are similar to those by Demes et al. [4] for *G. senegalensis *and provide statistical support for the observation that the quadriceps femoris is the dominant musculature group for leaping specialists (vertical clinger and leaper especially). 

In cheirogaleids, there is a greater balance of mass between musculature associated with propulsion (hip and knee extensors and ankle plantarflexors) and “other” muscles (Figures 3 and 4), as seen in the quadrupedal *Varecia variegata* [4]. There are subtle differences between the cheirogaleids; it is unclear if these are functionally significant. However, it may be noteworthy that the species' locomotor behavior is not described identically. *M. murinus *is described to employ leaping behaviors with great frequency [15], whereas *C. medius *has a generalized arboreal quadrupedal style of locomotion [15, 36]. 

### 4.4. Locomotor Behavior and Musculoskeletal Ontogeny

Infant primates are not immediately adept at locomotion, perhaps especially those with highly specialized modes [36]. At least, some leaping specialists are known to undergo postnatal proportional changes in the limbs and trunk [37]. Thus maturation of the skeletal system among species is of interest. Watts [10] argued that ossification sequences in the limbs are similar in many hominoids, New World monkeys, and prosimians (a term used here as a grade of primates). If true, the results on degree of ossification of the hip are unsurprising. Despite the different locomotor tendencies between cheirogaleids and galagids, and some locomotor differences within these families [15], all perinatal specimens were similar in the extent of ossification at the hip. Further work seems important. Very few prosimians have been studied regarding early skeletal maturation. In addition, our focus on the hip leaves unknown whether more distal joints vary in extent of ossification. 

Our sample provides more detailed information on ontogeny of hind limb muscle mass. Previously, Atzeva et al. [1] observed that among five species of prosimian primates, the ratio of total hind limb muscle mass/body mass is smaller in infants than in adults, suggesting primates are relatively poorly muscled at birth. The findings in this study support this observation. If external hip rotators are excluded (since these were not measured in all cases) the total hind limb muscle mass/body mass ratio in *C. medius *is 0.04 for adults and 0.01 for infants. In *G. moholi*, the ratio is 0.06 for adults and 0.02 for infants. The ratio in infants could actually be inflated, since one of the *G. moholi* specimens was two weeks old. If this represents a broad characteristic of primates, it suggests that one advantage for the relatively long dependency of infant primates [38, 39] is for hind limb muscular gain.

Our data on ontogenetic changes in relative muscle mass are based on a slightly larger sample than a previous report [1], allowing some additional interpretation. The increased sample of *C. medius *yielded a larger overall hind limb muscle mass for adults (5.95 g) and a smaller overall hind limb muscle mass for infants (0.01 g). Correspondingly, the adult/infant muscle group ratios is higher in this study (Table 1, see columns 3, 6) compared to those of Atzeva et al. [1] and is higher than the ratios for *G. moholi *for all muscle groups. This indicates a greater postnatal muscle mass gain in *C. medius.* This may be interpreted to indicate that a greater degree of altriciality is associated with relatively less muscle mass at birth. The relatively altricial *Varecia variegata *also had high ratios. 

Previously, musculoskeletal changes across age have been discussed in terms of how they relate to locomotor ontogeny [14, 37] particularly and how does ontogeny of locomotor anatomy relate to the transition from an unspecialized strategy (e.g., crawling) to the adult strategies, as observed in captive and wild leaping specialists [40]. Atzeva et al. [1] found some specializations are exhibited precociously at birth. Within hind limb segments, muscular mass distribution reflects adult locomotor behaviors. For example, leaping specialists tend to have proportionally large knee extensors in the thigh and perhaps large leg plantarflexors. Muscular mass is not distributed similarly *between *limb segments across age, however. Previous studies have noted a shift in limb muscle mass from distal to proximal segments [1, 14]. In one sense, this mass shift appears to correspond to a transition from predominantly grasping limb activities to locomotor activities in the limbs (see Raichlen, [14], for discussion). However, Atzeva et al. [1] noted that this shift occurs in all primates that they studied, including those that ride their mothers and those that are instead carried orally. Thus, an additional factor may underlie this proximal mass gain. 

The results of the present study may shed additional light on this issue, by showing a pronounced muscle mass shift to the thigh in a species that habitually uses leaping behaviors as adults. For *G. moholi*, at least, the reliance on knee extensors for leaping [4] makes the thigh an arguably critical segment for mass gain. An interesting question would be to determine if a species relying more extensively on the hip musculature for leaping (e.g., the sifaka) gains proportionally more mass in that segment. Interestingly, the species with the least specialized (here, meaning the most dedicatedly quadrupedal) locomotion as adults, *C. medius*, appears to show little or no change in proximal to distal percentage distribution of muscle mass between age groups. Thus, muscle mass gain to specific limb segments may be a critical milestone for primates with extremely specialized modes of locomotion. 

## Figures and Tables

**Table 1:** Muscle group mass (g) averages and ratios of hind limb musculature in adult and infant *C. medius *and *G. moholi. *

Muscle group^1^	*C. medius*			*G. moholi*		

	Adults	Infants		Adults	Infants	
	*n* = 4	*n* = 5	Ratio^1^	*n* = 4	*n* = 4	Ratio
	Average	Average	Average	Average	Average	Average
	(range)	(range)	(range)	(range)	(range)	(range)
Iliopsoas	0.38	0.004^2^	100.0	0.35	0.013	26.9
	(0.25–0.50)		(65.8–131.6)	(0.21–0.46)	(0.0130–0.0132)	(15.9–35.4)
Gluteals^3^	0.86	0.0125	68.8	0.65	0.020	32.5
	(0.41–1.21)	(0.005–0.019)	(21.6–242.0)	(0.43–0.86)	(0.016–0.023)	(18.7–53.8)
Small Hip Lat. Rotators	0.17	—	—	0.25	0.007	35.7
	(0.07–0.30)	—	—	(0.12–0.40)	(0.005–0.009)	(13.3–80.0)
hamstrings	0.98	0.011	89.0	1.35	0.040	33.8
	(0.31–1.58)	(0.005–0.014)	(21.8–343.7)	(0.52–1.92)	(0.025–0.055)	(9.40–76.6)
sartorius	0.14	0.002	70.0	0.50	0.008	62.5
	(0.11–0.18)	(0.001–0.003)	(33.2–361.0)	(0.03–1.70)	(0.006–0.011)	(2.5–267.3)
quadriceps	1.18	0.017	69.4	5.48	0.109	50.3
	(0.47–1.57)	(0.013–0.023)	(21.0–119.7)	(4.44–6.00)	(0.085–0.124)	(35.8–70.4)
hip adductors	0.68	0.008	85.0	0.98	0.018	54.4
	(0.28–1.26)	(0.003–0.013)	(22.6–369.6)	(0.46–1.83)	(0.017–0.019)	(24.8–106.1)
superficial ankle flexors	0.40	0.010	40.0	0.63	0.019	33.2
	(0.19–0.57)	(0.005–0.016)	(12.0–123.8)	(0.50–0.92)	(0.013–0.025)	(20.0–69.4)
deep ankle flexors	0.45	0.006	75.0	0.41	0.018	22.8
	(0.32–0.65)	(0.003–0.008)	(42.4–215.8)	(0.33–0.49)	(0.006–0.023)	(14.5–76.3)
anterior comp. leg	0.44	0.005	88.0	0.37	0.013	28.5
	(0.28–0.81)	(0.003–0.010)	(27.2–269.0)	(0.20–0.59)	(0.005–0.020)	(10.2–128.3)
lateral comp. leg	0.27	0.003	90.0	0.26	0.008	32.5
	(0.15–0.39)	(0.001–0.050)	(30.2–394.0)	(0.21–0.34)	(0.007–0.013)	(15.6–48.1)
Body mass^4^	156	12	13	180	13.4	13.4

^1^Average adult mm mass/average infant mm mass.

^2^This muscle could be reliably removed in only a single perinatal specimen of *C. medius.*

^3^gluteals: gluteus medius, gluteus minimus, gluteus superficialis ant., tensor fasciae femoris, iliopsoas; hamstrings: flexor cruris lateralis, semitendinosus, semimembranosus; quadriceps: rectus femoris, vastus intermedius, vastus medialis, vastus lateralis; adductors: pectineus, adductor brevis, adductor longus, adductor magnus (presemimembranosus*), gracilis; anterior compartment leg: tibialis anterior, extensor digitorum longus, extensor hallucis longus, abductor hallucis longus*; superficial flexors: gastrocnemius, soleus, plantaris; deep flexors: peroneotibialis, flexor fibularis, tibialis posterior, flexor tibialis; lateral compartment: peroneus brevis, peroneus longus, peroneus digiti quarti, peroneus digiti quinti,* (*, if present).

^4^data from Kappeler and Pereira [23].

**Table 2:** Mean percentage (SD) of functional muscle groups in adult primates with results of statistical tests.

	*Galago*	*Cheirogaleus*	*Microcebus*	Kruskal-Wallis test
% hip extensors (including hamstrings)	16.0^†^ (4.1)	27.0 (4.3)	30.0^†^ (1.7)	*χ* ^2^ = 8.86, *P* < .02
% knee extensors	50.0^†^ (7.0)	20.0 (2.9)	25.0^†^ (2.6)	*χ* ^2^ = 10.38, *P* < .01
% ankle plantarflexors	6.0^†^ (0.6)	7.0 (1.0)	7.0^†^ (0.6)	*χ* ^2^ = 7.4, *P* < .05

^†^Pairs of means that were significantly different using a Mann Whitney *U-*test with a sequential Bonferroni correction to assess significance [24]. Use of this statistical correction was extensively discussed by Cabin and Mitchell [28]. They noted that failure to use this correction inflates Type I errors (falsely rejecting the null hypothesis), while “overzealous use” of this correction inflates Type II errors (falsely accepting the null hypothesis. One possible approach would be to pool all post hoc tests for correction, in which case the range of corrected *P* values is  .006 to  .05. In this case, none of the pairs are significantly different, but the likelihood of Type II errors appears markedly increased. We applied the sequential Bonferroni correction separately for the three Mann Whitney *U-*tests that followed each Kruskal-Wallis test.

**Table 3:** Measurements of joint thickness (mm).

		*M. murinus*			*C. medius*			*G. moholi*		
		AC	SP	Ratio	AC	SP	Ratio	AC	SP	Ratio
Acetabulum	Mean	0.123	0.05	3.77	0.254	0.13	2.21	0.679	0.143	6.48
	(SD)	(0.055)	(0.046)		(0.049)	(0.069)		(0.328)	(0.173)	
	range	0.056–0.201	0.01–0.162		0.209–0.307	0.07–0.206		0.336–0.975	0.059–0.445	

Femur	Mean	0.155	0.056	4.99	0.135	0.031	5	0.297	0.084	6.40
	(SD)	(0.071)	(0.092)		(0.037)	(0.018)		(0.167)	(0.037)	
	range	0.061–0.265	0.02–0.375		0.11–0.178	0.015–0.051		0.113–0.423	0.015–0.533	
